# 59-year-old Woman with Acute Blindness

**DOI:** 10.5811/cpcem.66397

**Published:** 2026-10-26

**Authors:** Nicolas Contillo, Michael Sibel, Laura J Bontempo, John David Gatz

**Affiliations:** * University of Maryland Medical Center, Department of Emergency Medicine, Baltimore, Maryland; † University of Maryland School of Medicine, Department of Emergency Medicine, Baltimore, Maryland

## Abstract

When a patient with acute blindness presents to the emergency department (ED), the evaluation typically suggests one of the “can’t miss” diagnoses ingrained in emergency physicians during training. How does the differential change when the patient’s presentation does not neatly reveal an expected pathology? A 59-year-old woman presented to the ED with acute binocular blindness in the setting of three days of a flu-like illness. This case offers a practical approach to opening the lens on the presentation of acute blindness.

## CASE PRESENTATION (DR. CONTILLO)

A 59-year-old woman with a history of asthma, hypertension, type 2 diabetes mellitus with neuropathy, and seasonal allergies presented to the emergency department (ED) after she awoke with complete bilateral vision loss accompanied by nausea, emesis, and diarrhea. She reported three days of flu-like symptoms, including subjective fever, myalgias, nausea, diarrhea, and “dark” emesis. Similar symptoms were reported among other members of her household. Her vitals on arrival were as follows: blood pressure, 83/48 mm Hg; heart rate, 80 beats per minute; respiratory rate, 21 breaths per minute; SpO_2_, 100% on room air; and temperature, 31.6 °C.

The patient had been unable to tolerate oral medications for several days due to emesis and admitted to taking a “double dose” of her routine medications the previous day to compensate. She denied any illicit substance ingestion. Past surgical history included a cesarean section. Family history included hypertension and breast cancer. Her home medications were albuterol, cetirizine, fluticasone nasal spray, atorvastatin, gabapentin, metformin, tirzepatide, valsartan, metoprolol, vitamin D, and ferrous sulfate. She was allergic to iodinated contrast (anaphylaxis), lisinopril (angioedema), latex, and the COVID-19 vaccine.

Physical examination revealed a toxic-appearing woman in extremis. She had no external signs of trauma such as abrasions, lacerations, or ecchymoses. Her ocular exam was notable for complete binocular vision loss with 3-mm, round, nonreactive pupils bilaterally. There was no proptosis, lid edema, exudate, conjunctival injection, hyphema, or hypopyon. Her extraocular movements were intact. Neurologic exam was notable for full strength in all extremities, normal light touch sensation, and preserved cranial nerve function with the exception of cranial nerve II. Her mucous membranes appeared dry. Her neck was supple without signs of meningismus. Her heart sounds were normal without murmurs. Her lung fields were clear, and respiratory effort was normal. Her abdomen was soft, nondistended, with mild generalized tenderness and no rebound or guarding. Her extremities were cool to touch, with delayed capillary refill >3 seconds and thready radial and dorsalis pedis pulses. There was no edema nor were there abnormal dermatologic findings such as rashes or mottling.

Initial laboratory studies (see Table) demonstrated a severe elevated anion gap metabolic acidosis, low bicarbonate, and markedly elevated serum lactate. An electrocardiogram (ECG) was obtained (see Figure). Computed tomography (CT) imaging of the brain, chest, abdomen and pelvis was obtained without use of intravenous contrast due to her previously documented history of anaphylaxis to iodinated contrast. No acute abnormalities were observed.

## CASE DISCUSSION (DR. SIBEL)

Presented is a 59-year-old woman with a chief complaint of acute, complete blindness preceded by a flu-like illness with positive sick contacts in the home. Her symptoms were associated with vomiting of dark emesis along with diarrhea. She had been unable to tolerate her medications but reported that she took a “double dose” of her medications the day prior to her ED presentation.

When presented with a patient with acute blindness my first inclination is to consider an ocular problem. Diagnoses that come to mind are retinal detachment, central retinal artery occlusion, central retinal vein occlusion, and vitreous hemorrhage. While possible, these pathologies would not be expected to present with bilateral blindness. This opens the differential substantially, bringing intracranial and systemic processes into consideration. More posterior in the optic chain is the optic nerve and chiasm. Bilateral optic neuritis is possible but again typically presents unilaterally. Compression on the optic chiasm could cause a bilateral visual disturbance. Diagnoses here include pituitary adenoma, pituitary apoplexy, hypothalamic masses, and craniopharyngioma. Most of these diagnoses are indolent and would be expected to first present with visual field deficits prior to complete bilateral blindness. The final consideration involving the optic chain would be the vision center in the brain. Ischemic stroke and intracranial hemorrhage should be considered. Systemic processes that could cause bilateral blindness include toxic ingestions leading to toxic retinopathy, metabolic retinopathy, and severe retinal ischemia secondary to a shock state.

The patient’s past medical history was unremarkable and offered no clear indicators regarding the underlying etiology. Her history of diabetes and hypertension, however, raise thoughts to a potential thromboembolic condition such as an ischemic stroke or amaurosis fugax. Additionally, her diagnosis of diabetes could explain blindness in the setting of diabetic retinopathy (DR); however, I would have expected this to be a more indolent course. She denied substance use, making the likelihood of an unprescribed intoxicant such as methanol less likely. Her medications do not seem to be out of the ordinary for her medical history. However, it is notable that she is on tirzepatide, which is a glucagon-like peptide-1 receptor agonist (GLP-1RA). Recent literature has linked the use of medications like this to increased risk of new-onset non-arteritic anterior ischemic optic neuropathy (NAION) in addition to DR.^1^,^2^ While a more insidious course would be expected for DR, a sudden onset of vision loss could be explained by NAION. That, therefore, would make it easy to anchor on tirzepatide being the causative agent of the patient’s vision changes. However, this does not explain the whole clinical picture including the hypotension, hypothermia, acute kidney injury, and lactic acidosis, necessitating exploration of alternative etiologies for the patient’s blindness.

Her vital signs are most remarkable for hypotension and hypothermia. These two findings help to narrow the differential when considering them as being caused by the same disease process. They raise diagnoses such as sepsis or endocrine pathology (ie, hypothyroidism, adrenal crisis, hypoglycemia, and toxicological exposure) to the top of the differential. Hemorrhage and hypovolemic shock also remain on the differential given her history of diarrhea and dark emesis. Her respiratory status and normal oxygenation make obstructive shock pathologies, such as massive pulmonary embolism and tension pneumothorax, less likely. New-onset cardiogenic causes are also a consideration; however, hypothermia would not typically be expected with these diagnoses. A point-of-care ultrasound to assess her ejection fraction and inferior vena cava diameter would be useful in taking these off the differential.

The patient’s exam is most notable for complete binocular blindness with 3-mm nonreactive pupils. An intact pupillary light reflex requires a functional retina, optic nerve, brain stem, and cranial nerve III. Her normal level of consciousness eliminates brain stem dysfunction, and bilateral cranial nerve III deficits would be highly unlikely. This, therefore, leads me to believe that the source of blindness is noncortical and that the origin is somewhere in the retinas or optic nerves. Considering the patient’s presentation, high on the differential would be toxicologic/metabolic retinopathy, severe bilateral retinal ischemia, and pituitary apoplexy. Reassuringly, the remainder of her neurologic exam is normal removing a central nervous system cause of the patient’s presentation from the differential because bilateral blindness from central nervous system pathology would be expected to have other neurologic findings as well. Most diagnoses that I would be looking for in acute blindness such as retinal detachment, optic neuritis, posterior reversible encephalopathy syndrome, and idiopathic intracranial hypertension do not fit with the patient’s hemodynamic picture because these pathologies are more localized and are not known to present in a shock state.

Her labs were most notable for an indeterminant anion gap, lactic acidosis, and severe kidney injury. On further investigation, the anion gap is indeterminant due to a critically low bicarbonate that surpassed the tolerances of the lab equipment, giving a value of less than 5 millimoles per liter. Through manual calculation I can estimate her anion gap to be between 39 and 44, adding a profound elevated anion gap to her notable lab values. Her complete blood count (CBC) showed a significant leukocytosis but without a shift in cell lines, which could be explained by an acute phase reaction. This, in addition to relatively unremarkable imaging and essentially negative urinalysis (UA), moves sepsis lower on the differential. Her CBC showed a normal hemoglobin and hematocrit which, along with no source of bleeding, removes hemorrhage from the differential.

Other nonspecific lab abnormalities including elevated lipase and mildly elevated aspartate aminotransferase are less concerning for pancreatic and liver pathology in the setting of negative imaging and nonfocal abdominal tenderness. Of note, acetone and glucose were present in the UA, and she also had an elevated blood sugar, which raised the possibility of diabetic ketoacidosis (DKA). However, small acetone does not necessarily explain the significant anion gap. Early DKA is a consideration but does not explain the patient’s entire clinical presentation. Toxic alcohol exposure is excluded because her methanol and ethanol levels are negative. The patient’s thyroid stimulating hormone and thyroxine are normal, removing myxedema from the differential. Additionally, her complete metabolic panel does not show electrolyte abnormalities that would suggest pituitary apoplexy or adrenal pathology. Her potassium and phosphorus are elevated, which can be explained by renal insufficiency. Her ECG demonstrates a normal sinus rhythm with normal axis, intervals, and mildly peaked T waves, which would be consistent with her elevated potassium.

Her head CT imaging is negative. While this cannot completely rule out an acute ischemic process, it does, in addition to a normal neurological exam outside her vision loss, help to exclude a central neurologic process from the differential. Additionally, there is no large sellar mass to suggest pituitary apoplexy as a cause for her blindness.

With the differential substantially narrowed, what is left that could explain the patient’s bilateral blindness with hypotension, hypothermia, and severe lactic acidosis? For acute bilateral, noncortical blindness, severe bilateral retinal ischemia could be caused by systemic hypotension or toxic/metabolic retinopathy. We have ruled out most pathologies that can cause hypotension; however, severe lactic acidosis could be the culprit, further exacerbated by hypovolemia in the setting of vomiting and diarrhea. With hypothermia, drugs and toxicologic causes are the highest on the differential. With these differentials in mind, the most significant diagnostic findings of profound lactic acidosis and kidney injury can be used to narrow the possibilities. There are only a few things that can cause such profound lactic acidosis in an awake and alert patient. Potential etiologies include an ischemic process such as limb or bowel ischemia. However, the patient’s exam and CT imaging did not reveal any such process. The other is toxicologic, specifically toxins that disrupt metabolism. There is, however, no history to suggest that the patient encountered a significant toxic exposure, except, possibly through her prescribed medications.

In reviewing her medications, the one that stands out is metformin. Metformin inhibits oxidative phosphorylation and shifts cells to anaerobic metabolism. When toxic levels are reached, severe lactic acidosis can occur. This syndrome is known as metformin-associated lactic acidosis (MALA). While there is no history to suggest that she overdosed, she had taken a double dose of her medications the day prior. Typically, this would not be enough to reach toxic blood levels of metformin. Remembering the patient’s history, however, she reported that her blindness was preceded by three days of illness which was associated with nausea, vomiting, and diarrhea. This likely caused acute kidney injury (AKI) which would have affected metformin clearance, as metformin is mostly cleared renally. The “double dose” that she took in the setting of profound AKI likely led to toxic metformin levels. This is well documented as a cause of MALA. Her blindness could be explained by toxic metabolic pathology due to severe lactic acidosis. Considering all the above factors in a patient presenting with acute bilateral blindness, a serum metformin assay would be the diagnostic test of choice.

## CASE OUTCOME (DR. CONTILLO)

The patient’s metformin level ultimately resulted at 17 mcg/mL, well above the therapeutic range of 1–2 mcg/mL, confirming the diagnosis MALA. The patient was admitted to the medical intensive care unit and initiated on high-intensity continuous renal replacement therapy (CRRT) within hours of arrival. She required transient vasopressor support, which was discontinued within 24 hours as the acidosis resolved. Neurologic function and vision fully recovered by hospital day six. She was ultimately discharged home in stable condition with transition to an insulin-based regimen for diabetes management.

## RESIDENT DISCUSSION (DR. CONTILLO)

Metformin, a biguanide derived from *Galega officinalis* (goat’s rue), was first described in 1922 and approved by the US Food and Drug Administration for use in 1994. It acts primarily via stimulation of the adenosine monophosphate-activated protein kinase pathway, improving insulin sensitivity and decreasing hepatic gluconeogenesis.^3^ Toxicity occurs through inhibition of mitochondrial cytochrome complexes, leading to failure of oxidative phosphorylation. As a result, pyruvate metabolism shifts toward lactate production and reactive oxygen species accumulation, resulting in profound metabolic acidosis. MALA typically arises in the setting of renal impairment, overdose, or other hypoperfusive states such as septic or cardiogenic shock.^4^

Clinical features of MALA may include nausea, vomiting, abdominal pain, hemodynamic collapse, and altered mental status. Presentation with complete bilateral blindness, as in this case, is exceedingly rare, although several case reports exist in the literature. While the exact mechanism is unclear, acidosis-induced dysfunction of retinal photoreceptor ion transmission is suspected to play a role in this phenomenon.^5^

The differential diagnosis for MALA includes septic shock, cardiogenic shock, bowel ischemia, DKA, combined MALA-DKA, and acute hepatic failure. Given its nonspecific findings, diagnosis relies on a combination of historical context, lactic acidosis, and elevated serum metformin concentrations, though this laboratory analysis is rarely immediately available to the emergency physician.

The Extracorporeal Treatments in Poisoning (EXTRIP) workgroup has set forth guidelines for the management of MALA.^6^ Mild cases may respond to volume resuscitation, correction of underlying causes, vasopressor support, and sodium bicarbonate therapy. Severe MALA—defined by lactate greater than 20 mmol/L, pH less than 7.0, hemodynamic instability, or end-organ dysfunction—requires urgent extracorporeal removal via CRRT or intermittent hemodialysis. Extracorporeal therapy should continue until lactate decreases to less than 3 mmol/L and pH increases to greater than 7.35.

This case highlights the importance of considering MALA in the differential diagnosis for acute visual loss associated with shock and metabolic acidosis in diabetic patients. Early recognition and prompt initiation of renal replacement therapy can be lifesaving, and in this case lead to complete recovery. For emergency physicians, awareness of this rare but critical diagnosis remains essential in identifying at-risk patients and initiating guideline-based management.

## FINAL DIAGNOSIS

The final diagnosis is transient blindness from metformin-associated lactic acidosis.

## KEY TEACHING POINTS

1. Metformin-associated lactic acidosis can present with chief complaints of neurologic and visual manifestations.
2. In a diabetic patient with volume depletion, AKI, and elevated lactate; MALA should be considered even without an obvious overdose history.
3. A normal noncontrast CT and a nonfocal neurologic exam do not exclude a metabolic diagnosis. Toxic-metabolic etiologies should be considered in patients with abnormal neurologic findings.
4. Early renal replacement therapy is lifesaving in severe MALA. The EXTRIP guidelines recommend extracorporeal treatment for severe toxicity, with continuation until lactate is less than 3 mmol/L and pH is greater than 7.35.

## Figures and Tables

**Figure:**
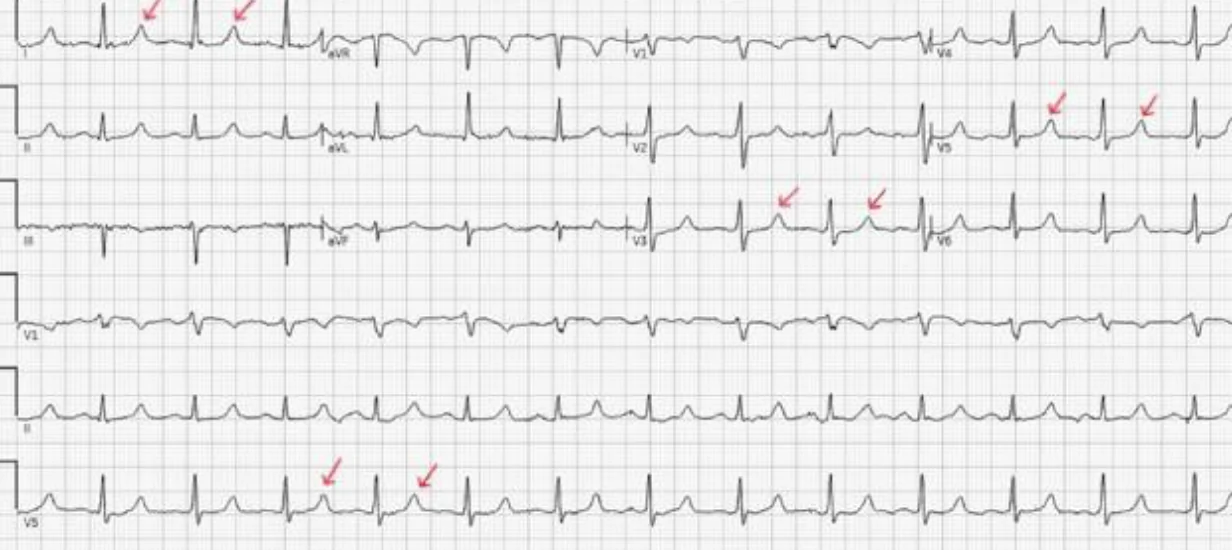
Electrocardiogram of a 59-year-old woman with acute blindness. Red arrows highlight peaked T waves.

**Table 1:** Laboratory results of a 59-year-old woman with acute blindness.

Test Name	Patient Value	Reference Range
Complete blood count		
White blood cell	23.8 K/mcL	4.5–11 K/mcL
Hemoglobin	11.0 g/dL	11.9–15.7 g/dL
Hematocrit	36.6%	35.0–45.0%
Platelets	304 K/mcL	153–367 K/mcL
Neutrophils	61.2%	40.0–70.0%
Lymphocytes	21.8%	20.0–45.0%
Eosinophils	0.4%	0.0–6%
Coagulation studies		
Prothrombin time	19.2 seconds	11–13.5 seconds
International normalized ratio	1.5	0.8–1.2
Serum chemistries		
Sodium	137 mmol/L	136–145 mmol/L
Potassium	6.6 mmol/L	3.5–5.1 mmol/L
Chloride	93 mmol/L	98–107 mmol/L
Bicarbonate	<5 mmol/L	21–30 mmol/L
Blood urea nitrogen	60 mg/dL	7–17 mg/dL
Creatinine	11.54 mg/dL	0.52–1.04 mg/dL
Glucose	283 mg/dL	70–100 mg/dL
Albumin	5.0 g/dL	3.2–4.6 g/dL
Anion gap	Indeterminate*	4–12 mEq/L
Magnesium	2.5 mg/dL	1.6–2.6 mg/dL
Phosphorus	19.0 mg/dL	2.5–4.5 mg/dL
Lactate	>17 mmol/L	0.5–2.0 mmol/L
Hepatic studies		
Total bilirubin	1.2 mg/dL	0.3–1.2 mg/dL
Aspartate aminotransferase	80 units/L	14–36 units/L
Alanine aminotransferase	45 units/L	0–34 units/L
Alkaline phosphatase	68 units/L	38–126 units/L
Venous Blood Gas		
pH	<7.00	7.31–7.41
pCO_2_	19 mmHg	41–51 mmHg
pO_2_	64 mmHg	30–50 mmHg
Additional Labs		
Lipase	2,948 U/L	10–60 U/L
Ethanol level	<10 mg/dL	<10.0 mg/dL
Methanol level	<5 mg/dL	<5 mg/dL
Acetone	Small	Negative
Thyroid stimulating hormone	0.4 mIU/L	0.4–4.5 mIU/L
Free thyroxin	1.1 ng/dL	0.8–1.8 ng/dL
Serum osmolality	349 mOsm/kg	275–295 mOsm/kg
Urinalysis studies		
White blood cell count	0 – 2 per hpf	0–5 per hpf
Red blood cell count	3 – 5 per hpf	0–2 per hpf
Leukocyte esterase	negative	negative
Nitrite	negative	negative
Bacteria	negative	negative
Urine pregnancy	negative	negative
Glucose	2+	negative
Ketones	1+	negative

* Unable to calculate due to unmeasurable bicarbonate level.

*dL*, deciliter; *g*, grams; *hpf*, high-power field; *K*, thousands; *kg*, kilogram; *L*, liter; *mcL*, microliter; *mEq*, milliequivalent; *mg*, milligram; *mIU*, milli-international units; *mmol*, millimole; *mmHg*, millimeters of mercury; *mOsm*, milliosmole; *ng*, nanogram; *pCO_2_*, partial pressure of carbon dioxide; *pH*, potential of hydrogen; *pO_2_*, partial pressure of oxygen; *U*, units.
